# Hypoxia Downregulates MAPK/ERK but Not STAT3 Signaling in ROS-Dependent and HIF-1-Independent Manners in Mouse Embryonic Stem Cells

**DOI:** 10.1155/2017/4386947

**Published:** 2017-07-27

**Authors:** Jan Kučera, Julie Netušilová, Stanislava Sladeček, Martina Lánová, Ondřej Vašíček, Kateřina Štefková, Jarmila Navrátilová, Lukáš Kubala, Jiří Pacherník

**Affiliations:** ^1^Institute of Experimental Biology, Faculty of Science, Masaryk University, Kotlářská 267/2, 61137 Brno, Czech Republic; ^2^Institute of Biophysics, Academy of Sciences of the Czech Republic, Královopolská 2590/135, 61200 Brno, Czech Republic; ^3^International Clinical Research Center, Centre of Biomolecular and Cellular Engineering, St. Anne's University Hospital, Pekařská 53, 65691 Brno, Czech Republic

## Abstract

Hypoxia is involved in the regulation of stem cell fate, and hypoxia-inducible factor 1 (HIF-1) is the master regulator of hypoxic response. Here, we focus on the effect of hypoxia on intracellular signaling pathways responsible for mouse embryonic stem (ES) cell maintenance. We employed wild-type and HIF-1*α*-deficient ES cells to investigate hypoxic response in the ERK, Akt, and STAT3 pathways. Cultivation in 1% O_2_ for 24 h resulted in the strong dephosphorylation of ERK and its upstream kinases and to a lesser extent of Akt in an HIF-1-independent manner, while STAT3 phosphorylation remained unaffected. Downregulation of ERK could not be mimicked either by pharmacologically induced hypoxia or by the overexpression. Dual-specificity phosphatases (DUSP) 1, 5, and 6 are hypoxia-sensitive MAPK-specific phosphatases involved in ERK downregulation, and protein phosphatase 2A (PP2A) regulates both ERK and Akt. However, combining multiple approaches, we revealed the limited significance of DUSPs and PP2A in the hypoxia-mediated attenuation of ERK signaling. Interestingly, we observed a decreased reactive oxygen species (ROS) level in hypoxia and a similar phosphorylation pattern for ERK when the cells were supplemented with glutathione. Therefore, we suggest a potential role for the ROS-dependent attenuation of ERK signaling in hypoxia, without the involvement of HIF-1.

## 1. Introduction

Embryonic development in its early stages takes place in a hypoxic microenvironment, and oxygen (O_2_) has a significant impact on cellular differentiation or cell fate decisions [1]. ES are derived from preimplantation blastocyst, and hypoxia is thus suggested to modify cellular differentiation and regulate pluripotency. However, the outcome of stem cell cultivation in reduced O_2_ tension remains highly controversial [2–5].

Cellular response to hypoxia is primarily orchestrated by hypoxia-inducible factor (HIF). HIF is a heterodimeric protein belonging to a family of environmental sensors known as a bHLH-PAS (basic-helix-loop-helix-Per-Arnt-Sim) transcription factors. HIF consists of a constitutively expressed HIF-*β* subunit and O_2_-regulated HIF-*α* subunit [6]. Three *α* isoforms termed HIF-1*α*, HIF-2*α,* and HIF-3*α* are currently described [7]. When O_2_ is not a limiting factor, the HIF-*α* subunit is rapidly hydroxylated by the family of proline hydroxylases (PHD) and targeted for subsequent proteasomal degradation [8–10]. Hypoxia inactivates PHD leading to the accumulation of the *α* subunit. After dimerization with the *β* subunit in the nucleus, HIF binds to a conserved DNA sequence known as the hypoxia-responsive element (HRE) to transactivate a myriad of hypoxia-responsive genes. HIF-1 heterodimer consisting of HIF-1*α* and HIF-*β* is the most important for cell adaptation to hypoxia [11]. Notably, O_2_-dependent hydroxylation is implicated also in the modification of other (non-HIF) targets, and O_2_ is also a substrate for other various enzymes such as NADPH oxidases and monooxygenases [12]. Further, sensing and cellular response to low O_2_ levels are also associated with the modulation of reactive oxygen species formation (ROS) and alterations in the metabolism of mitochondria, including ATP production [13]. A particular cellular response to reduced O_2_ levels is thus complex and might be mediated in both HIF-dependent and -independent fashion.

The self-renewal and differentiation of murine ES cells is regulated by several signaling pathways. Among others, the signal transducer and activator of transcription 3 (STAT3) and phosphatidylinositol-4,5-bisphosphate-3-kinase/Akt (PI3K/Akt) signaling maintain ES cells in an undifferentiated pluripotent state [14, 15]. Conversely, mitogen-activated protein kinase/extracellular signal-regulated kinase (MAPK/ERK) signaling promotes the differentiation of ES cells [16]. Here, we focus on changes elicited in the activity of the mentioned signaling pathways in wild-type and HIF-1*α* deficient ES cells upon 24 h cultivation in 1% O_2_. The activity of these signaling pathways is regulated through phosphorylation and by an opposing process of dephosphorylation mediated by phosphatases. DUSPs are the MAPK-specific phosphatases that impede the activity of ERK and also stress-activated kinases. DUSP1, DUSP5, and DUSP6 are suggested as hypoxia-sensitive and responsible for ERK downregulation [17, 18]. Further, serine/threonine PP2A regulates both the MAPK/ERK and Akt pathways, and its modulation by hypoxia is proposed in various models [19, 20].

Our study demonstrates the sensitivity of ES cells to chronic hypoxia in the context of the dephosphorylation of ERK and Akt with the possible involvement of ROS-dependent mechanisms. In agreement with other studies [21–23], we suggest that cultivation in 1% O_2_ is in our system also associated with the decline of markers associated with the undifferentiated status of ES cells, despite the impairment of prodifferentiation ERK signaling.

## 2. Materials and Methods

### 2.1. Cell Culture and Treatment

Feeder-free R1-adapted mouse ES cells (wild-type HIF-1*α* +/+) and HIF-1*α* −/− (a generous gift from Professor Carmeliet, Katholieke Universiteit Leuven, Belgium) [24] were propagated as described previously [25] in an undifferentiated state by cell culturing on tissue culture plastic coated with gelatin (0.1% porcine gelatin solution in water) in Dulbecco's modified Eagle's medium (DMEM) containing 15% fetal bovine serum (FBS), 100 IU/ml penicillin, 0.1 mg/ml streptomycin, and 1× nonessential amino acid (all from Gibco-Invitrogen, UK) and 0.05 mM *β*-mercaptoethanol (*β*-ME; Sigma-Aldrich, USA), supplemented with 1000 U/ml of leukemia inhibitory factor (LIF, Chemicon, USA), referred to here as the complete medium. Cells were seeded and after 24 h were cultivated in normoxia or hypoxia for 24 h, unless specified otherwise. Normoxia was defined as 95% ambient air and 5% CO_2_, hypoxia as 1% O_2_, 94% N_2_, and 5% CO_2_.

Cobalt (II) chloride (CoCl_2_), desferoxamine (DFO), dimethyloxalylglycine (DMOG), okadaic acid (OA), and glutathione (GSH) were provided by Sigma-Aldrich, USA. JNJ-42041935 (JNJ) was generously provided by Terry Barrett, Ph.D. (Johnson & Johnson Pharmaceutical Research & Development).

### 2.2. Cell Transfection and Luciferase Reporter Assay

Cell transfection and luciferase reporter assay were performed as described elsewhere [22]. Briefly, for the luciferase reporter assay, cells were transfected using polyethyleneimine (PEI) in a stoichiometric ratio of 4 *μ*l per 1 *μ*g of DNA 24 h after seeding.

0.5 *μ*g of pT81/HRE-*luc* construct containing three tandem copies of the erythropoietin HRE in front of the herpes simplex thymidine kinase promoter and the luciferase gene, expression vectors for murine HIF-1*α* and HIF-2*α* under cytomegalovirus promoter, and GFP [26] (all generously provided by Professor L. Poellinger, Karolinska Institutet, Sweden), APRE-luciferase gene reporter plasmid (STAT3-responsive acute-phase response element, luciferase reporter; APRE-*luc* [27]; kindly provided by Professor A. Miyajima, Institute of Molecular and Cellular Biosciences, University of Tokyo, Japan) and *Renilla* luciferase construct (Promega, USA) were used per one well in a 24-well plate. The cell culture medium was changed 6 h after transfection. For experiments involving APRE-*luc*, the medium was exchanged for fresh complete medium or medium without LIF. Dual-Luciferase Assay Kit (Promega, USA) was used for the evaluation of luciferase activity according to the manufacturer's instructions. Relative luciferase units were measured using a ChameleonTM V plate luminometer (Hidex, Finland) and normalized to the *Renilla* luciferase expression.

### 2.3. Small Interfering RNA (siRNA) Transfection

Cells were transfected by commercially available siRNA against DUSP1 (sc-35938), DUSP5 (sc-60555), DUSP6 (sc-39001) transcripts (each consisting of a pool of 3 target-specific 19-25 nt siRNAs designed to knock down gene expression), or related nonsilencing control (all Santa Cruz Biotechnology, USA) using Lipofectamine RNAiMAX Reagents (Thermo Fisher Scientific Inc., USA) according to the manufacturer's instructions. Cells were harvested at the indicated time, and the expression of particular DUSP transcripts was assessed by qRT-PCR, or the expressions of selected proteins and posttranslational modification were analyzed by western blot.

### 2.4. Quantitative Real-Time RT-PCR (qRT-PCR) Analysis

Total RNA was extracted using the UltraClean Tissue & Cells RNA Isolation Kit (MO BIO Laboratories, USA). Complementary DNA was synthesized according to the manufacturer's instructions for M-MLV reverse transcriptase (Sigma-Aldrich); 0.5 *μ*g of total RNA was used for cDNA synthesis. qRT-PCR was performed in a Roche Light-Cycler 480 instrument using LightCycler SYBRE Green Master Mix (Roche, Germany) according to the manufacturer's instructions. The primers, appropriate annealing temperatures, and PCR product lengths for the determined transcripts are listed in Table 1. The gene expression of each sample was expressed in terms of the threshold cycle normalized to the mean of *β*-actin and TATA-box binding protein (TBP) expression.

### 2.5. Western Blot Analysis

Western blot analysis and cell sample harvesting and preparation were performed by a standard procedure as presented previously [28]. We used primary antibodies against HIF-1*α* (GeneTex, USA), HIF-2*α*, Nanog (Novus Biologicals, USA), *β*-actin, Oct4 (Santa Cruz), p-Akt (S473), Akt, p-STAT3 (Y705), STAT3, p-ERK (T202/Y204), ERK, p-glycogen synthase kinase 3 (S9) (p-GSK3*β*), RAF1, p-RAF1 (S259), p-RAF1 (S338), early growth response protein 1 (EGR1), DUSP6, p-MAP/ERK (S217/221) (p-MEK), and MEK (all Cell Signaling Technology, USA). Following immunodetection, each membrane was stained by Amido black to confirm the transfer of the protein samples. The total level of *β*-actin was detected as loading control. Typical representative western blots from at least three independent experiments are shown. Densitometry analysis was performed using ImageJ software (NIH, USA) and relative protein expression was calculated after normalization to the total protein of interest or *β*-actin. Data are presented as the mean + SEM.

### 2.6. High-Performance Liquid Chromatography (HPLC) Analysis of ROS Production

The HPLC detection of O_2_^−^ was based on the detection of a specific product, 2-hydroxyethidium 2-OH-E(+), which is formed in the reaction of O_2_^−^ with HE as described previously [25, 29, 30]. Besides specific 2-OH-E(+), also a nonspecific product of hydride acceptors in reaction with HE, ethidium (E(+)), was detected. Briefly, the cells were seeded onto 60 mm tissue culture dishes 24 h before treatment. The cells were exposed to hypoxia for 24 h or treated with 10 mM GSH for 60 minutes. Thirty minutes before the end of the experiment, HE (Sigma-Aldrich, USA) in a final concentration of 10 *μ*M was added to the cells. The cells were washed two times with ice-cold PBS. To extract the HE products, ice-cold methanol was added to the cells for 15 minutes at 4°C in the dark and shaken. The supernatant was transferred to an Eppendorf tube and centrifuged. A 75 *μ*l sample was injected into the HPLC system (Agilent series 1100) equipped with fluorescence and UV detectors (Agilent series 1260) to separate the 2-OH-E(+) and E(+) products. Fluorescence was detected at 510 nm (excitation) and 595 nm (emission); the mobile phase consisted of H_2_O/CH_3_CN. A Kromasil C18 (4.6 mm × 250 mm) column was used as the stationary phase.

### 2.7. Alkaline Phosphatase (ALP) Activity Determination

ALP activity determination was performed by a standard procedure as presented previously [31, 32]. Briefly, the cells were seeded 24 h before treatment and exposed to hypoxia or normoxia for 24 h. After the incubation, the cells were washed twice with PBS and lysed in ALP assay lysis buffer (50 mM Tris pH 7.4, 150 mM NaCl, 1 mM EDTA, and 0.5% NP40; all components Sigma-Aldrich, USA). Protein concentration was determined using DC protein assay (Bio-Rad, USA) kit according to manufacturer's instructions. 5 *μ*g of protein was incubated in a 96-well plate (four parallel wells in each group) at 37°C with ALP substrate (4-p-nitrophenylphosphate; Sigma-Aldrich, USA) for 30 minutes. The reaction was stopped by adding 3 M NaOH and the optical densities were measured at 405 nm (620 nm reference) using a microplate reader (Hidex, Finland).

### 2.8. Statistical Analysis

Data are expressed as mean + standard error of the mean (SEM). Statistical analysis was assessed by *T* test or by one-way analysis of variance ANOVA and Bonferroni's Multiple Comparison posttest. Values of *P* < 0.05 were considered statistically significant (^∗^*P* < 0.05)

## 3. Results

### 3.1. Hypoxic Response in Wild-Type and HIF-1*α*-Deficient ES Cells

**Figure 1:**
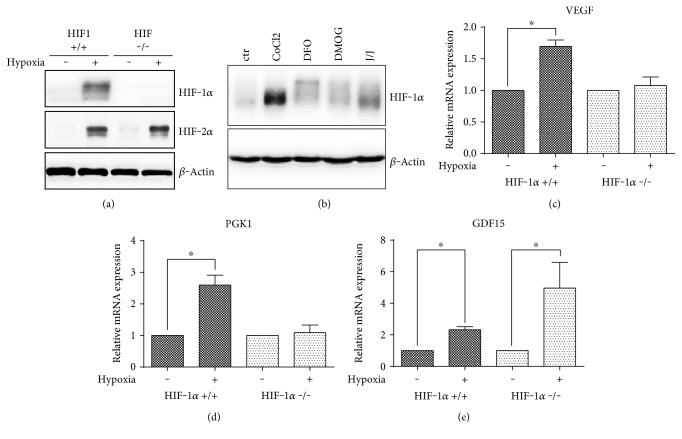
Stabilized HIF-1*α* and HIF-2*α* in hypoxia (24 h, 1% O_2_) in parental wild-type (HIF-1*α* +/+) and HIF-1*α* −/− ES cells (a) and pharmacologically stabilized HIF-1*α* in wild-type ES cells (b) determined by western blot. Total level of *β*-actin was used as a loading control. Relative expressions of HIF-dependent ((VEGF (c), PGK1 (d)) and -independent (GDF15 (e)) genes in normoxia and hypoxia in wild-type and HIF-1*α* −/− ES cells. Data are presented as mean + SEM from at least three independent experiments. Statistical significance was determined by *T* test (^∗^*P* < 0.05).

To determine the stabilization of HIF-1*α* and its role in hypoxic response in our system, we cultivated wild-type and HIF1*α*-deficient ES cells in normoxia and in the presence of 1% O_2_ for 24 h. In parallel, ES cells were also treated by hypoxia mimetics for 24 h (CoCl_2_–0.3 mM, DFO–0.05 mM, DMOG–3 mM, and JNJ–0.2 mM); concentrations were selected according to a comprehensive literature search and previous study [22]. As anticipated, exposure to hypoxia led to the stabilization of HIF-1*α* in wild-type but not in HIF-1*α* −/− ES cells. The level of HIF-2*α* in hypoxia-treated cells was equal in wild-type and HIF-1*α* −/− ES cells (Figure 1(a)). In a similar manner, treatment with hypoxia mimetics also resulted in HIF-1*α* stabilization with a certain variability in the efficacy of the employed compounds and with CoCl_2_ having the most pronounced effect (Figure 1(b)). The expressions of HIF-dependent (phosphoglycerate kinase 1 (PGK1), vascular endothelial growth factor (VEGF)), and HIF-independent (growth differentiation factor 15, GDF15) transcripts were determined by qRT-PCR. The increased expression of HIF downstream target genes (VEGF, PGK1) occurred in wild-type ES cells after 24 h hypoxic cultivation. The loss of HIF-1*α* reduced hypoxia-induced expression in both VEGF and PGK1 (Figures 1(c) and 1(d), resp.). The transcription of GDF15 was upregulated in both wild-type and HIF1*α* −/− cells cultivated in hypoxia (Figure 1(e)).

### 3.2. Hypoxia but Not HIF Stabilization Decreases ERK Signaling

**Figure 2:**
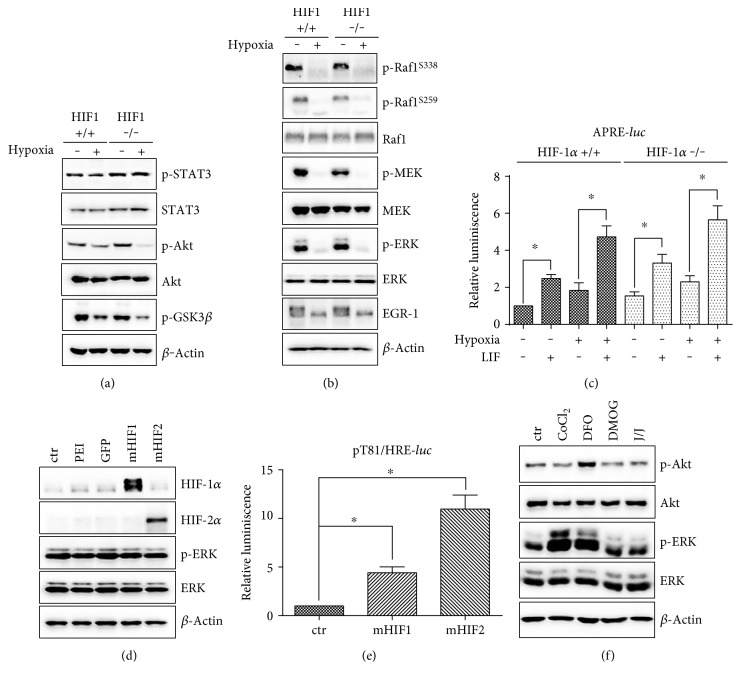
Effect of hypoxia on STAT3 and Akt (a) and ERK signaling (b) in wild-type and HIF-1*α* −/− ES cells. Analysis of STAT3 transcription activity (APRE-*luc*) in wild-type or HIF-1*α* −/− ES cultivated in complete medium (LIF) or LIF-free medium in normoxia or hypoxia (c). Effect of overexpression of mHIF1 and mHIF2 on ERK phosphorylation in wild-type ES cells (d). Analysis of HIF transcription activity (*pt81/HRE-luc*) induced by overexpression of mHIF-1 and mHIF-2 determined by luciferase reporter assay in wild-type ES cells (e). Effect of pharmacologically induced hypoxia on ERK phosphorylation in wild-type ES cells (f). The total level of *β*-actin was used as a loading control for western blot analyses. Data from luciferase assays are presented as mean + SEM from at least three independent experiments. Statistical significance was determined by one-way analysis of variance ANOVA and post hoc Bonferroni's multiple comparison test (^∗^*P* < 0.05).

The cultivation of ES cells in complete medium (supplemented with serum and LIF) results in the activation of several pathways that are responsible for ES cell maintenance. In normoxia, STAT3, Akt, and MAPK/ERK pathways are phosphorylated and, hence, active. Here, we aimed to assess how incubation in 1% O_2_ affects the phosphorylation of these signaling components. The phosphorylation of STAT3 remained unchanged by hypoxia. In contrast, the phosphorylation of Akt and its downstream target GSK3*β* at inhibitory serine 9 was decreased in hypoxia-exposed ES cells (Figure 2(a)). Notably, we observed a strong decrease in the phosphorylation of ERK and its upstream kinase MEK. The dephosphorylation of RAF1 was also apparent at both inhibitory S259 and activating S338 residues (Figure 2(b)). The attenuation of ERK signaling correlated with the downregulation of its downstream target EGR1 (Figure 2(b)). Dephosphorylation was not dependent on HIF-1*α* in any of the examined proteins.

To further analyze STAT3 signaling, we employed APRE-*luc*, a luciferase reporter system that responds to the transcriptional activity of STAT3. Wild-type and HIF-1*α* −/− ES cells were transfected with APRE-*luc* and, after media change, were further cultivated in the presence of LIF-free medium or complete medium for 24 h in either normoxia or hypoxia. APRE-*luc* activity was significantly upregulated in an HIF-independent manner in the presence of LIF, which is a known inductor of STAT3 signaling. This effect was preserved also in hypoxia (Figure 2(c)).

Next, we assessed the effect of HIF stabilization mediated via exogenous HIF-1 or HIF-2 expression. ES cells were transiently transfected by the vectors constitutively expressing mHIF-1*α* or mHIF-2*α*. Cells treated with PEI and transfected with GFP expression vector served as a control. We observed stabilization and increased levels of HIF-1*α* and HIF-2*α*, as well as the upregulation of HIF-mediated transcription activity; however, the phosphorylation of ERK kinase remained unaffected in both cases (Figures 2(d) and 2(e)).

The administration of the hypoxia mimetics CoCl_2_, DFO, DMOG, and JNJ for 24 h did not mimic the effect of hypoxia in the sense of ERK and Akt dephosphorylation. The phosphorylation of ERK kinase was upregulated following CoCl_2_ and DFO treatment and remained unaffected after the addition of DMOG and JNJ. Phosphorylation of Akt was upregulated by DFO and remained unchanged following treatment with other mimetics (Figure 2(f)).

### 3.3. Hypoxia Upregulates DUSP1 but DUSP6 Has the Most Prominent Effect on ERK Dephosphorylation

**Figure 3:**
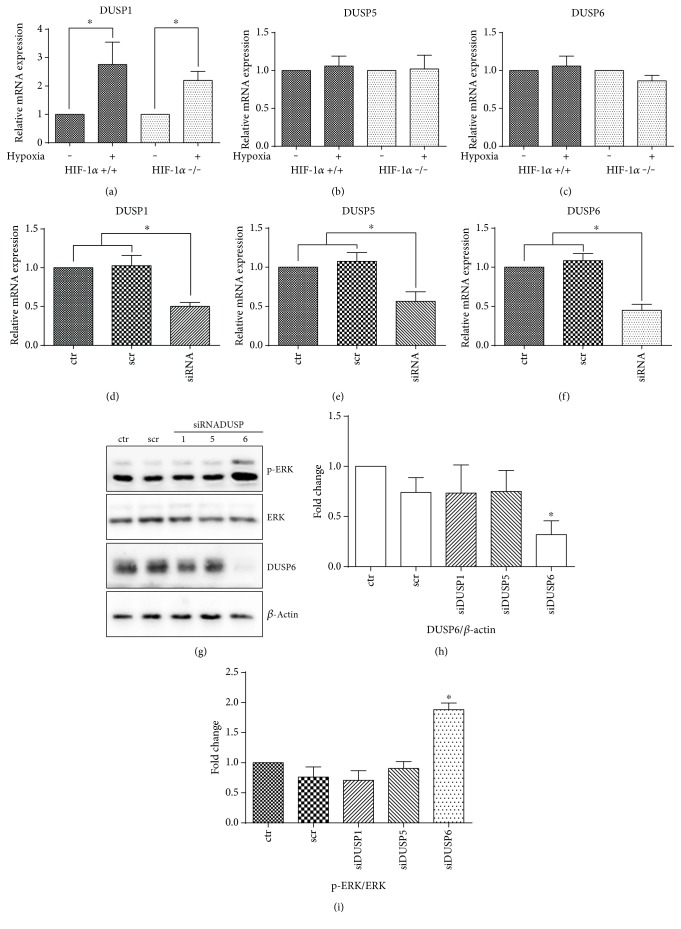
Relative expressions of DUSP1 (a), DUSP5 (b), and DUSP6 (c) in hypoxia in wild-type and HIF-1*α* −/− ES cells. Statistical significance was determined by *T* test (^∗^*P* < 0.05). Effect of siRNA-mediated silencing on DUSP1 (d), DUSP5 (e), and DUSP6 (f) expression in wild-type ES cells determined by qRT-PCR. Data are presented as mean + SEM from at least three independent experiments. Statistical significance was determined by one-way analysis of variance ANOVA and post hoc Bonferroni's multiple comparison test (^∗^*P* < 0.05). Effect of DUSP1, 5, and 6 siRNAs on ERK phosphorylation and DUSP6 levels in wild-type ES cells (g). The total level of *β*-actin was used as a loading control. Densitometry analysis of western blots expressed as fold change in DUSP6 protein expression (h) or ERK phosphorylation (i). Data are presented as mean + SEM from at least three independent experiments. Statistical significance was determined by one-way analysis of variance ANOVA and post hoc Bonferroni's multiple comparison test (^∗^*P* < 0.05).

Further, we investigated the role of DUSP phosphatases in hypoxia-driven ERK dephosphorylation. Firstly, the effect of hypoxia on DUSP1, 5, and 6 expressions was evaluated by qRT-PCR. The exposure of ES cells to hypoxia for 24 h increased DUSP1 expression (Figure 3(a)). Similar results were obtained using wild-type or HIF-1*α* deficient cells. In contrast to DUSP1, the level of transcripts of DUSP5 and 6 remained unaffected in hypoxia, again independently of the presence of HIF-1*α* (Figures 3(b) and 3(c), resp.). Next, we addressed the question of which DUSP has the most prominent effect on ERK dephosphorylation. We employed the RNA silencing of selected members of the DUSP family and screened for ERK phosphorylation. The transfection of ES cells by siRNA against DUSP1, 5, or 6 resulted in their decreased expression (Figures 3(d), 3(e), and 3(f), resp.). In the next step, we analyzed the effect of DUSP silencing on ERK phosphorylation status. Only the silencing of DUSP6 had a prominent effect on augmenting ERK phosphorylation. It was also correlated with the downregulation of DUSP6 protein (Figures 3(g), 3(h), and 3(i)).

### 3.4. Hypoxia Downregulates ERK Phosphorylation Regardless of DUSP6 Silencing

**Figure 4:**
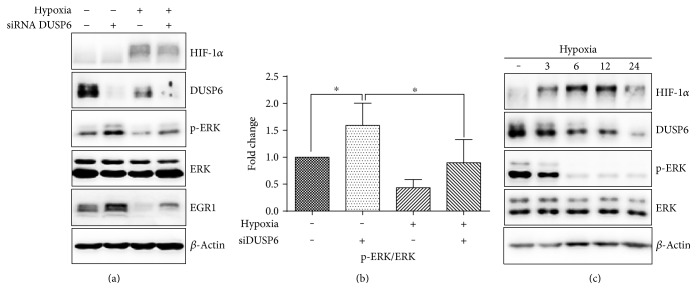
Effect of DUSP6 siRNA silencing on ERK signaling in normoxia and hypoxia in wild-type ES cells (a). Densitometry analysis of western blot expressed as fold change in ERK phosphorylation (b). Data are presented as mean + SEM from at least three independent experiments. Statistical significance was determined by one-way analysis of variance ANOVA and post hoc Bonferroni's multiple comparison test (^∗^*P* < 0.05). Effect of 3 h, 6 h, 12 h, and 24 h 1% O_2_ cultivation on DUSP6 protein expression and ERK phosphorylation in wild-type ES cells (c). Total level of *β*-actin was used as a loading control.

The RNA silencing of DUSP6 elevated the phosphorylation of ERK both in normoxia and hypoxia; however, increase in ERK phosphorylation following the DUSP6 silencing in hypoxia was not statistically significant (Figures 4(a) and 4(b)). Neither hypoxia-induced ERK dephosphorylation nor the reduction in EGR1 level were abolished following the DUSP6 silencing, as determined by western blot (Figure 4(a)). DUSP6 protein and ERK level were also determined in hypoxic conditions. Cells were cultured in 1% O_2_ for 3, 6, 12, and 24 h. Western blot analysis revealed the hypoxia-mediated downregulation of DUSP6 protein in ES cells at all examined time points. Similarly, ERK phosphorylation was also progressively downregulated in hypoxia (Figure 4(c)).

### 3.5. Okadaic Acid Partially Rescues Hypoxia-Mediated Dephosphorylation

**Figure 5:**
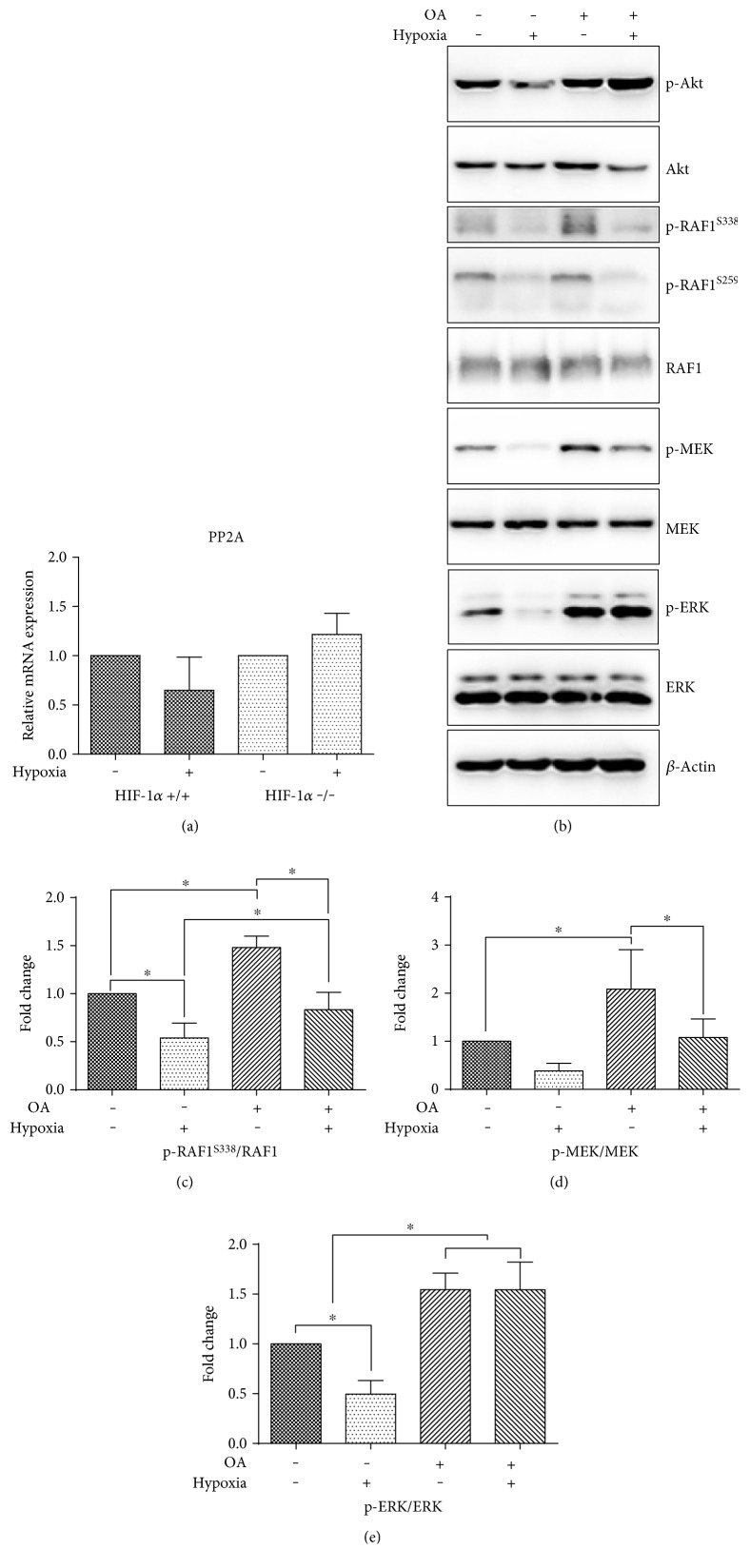
Relative expression of PP2A (a) in hypoxia in wild-type and HIF-1*α* −/− ES cells determined by qRT-PCR. Data are presented as mean + SEM from at least three independent experiments. Statistical significance was determined by *T* test. Effect of OA on signaling in hypoxia and normoxia in wild-type and HIF-1*α* −/− ES cells (b). Total level of *β*-actin was used as a loading control. Densitometry analysis of MAPK/ERK western blot expressed as fold change in RAF (S338) (c) MEK (d), and ERK phosphorylation (e). Data are presented as mean + SEM from at least three independent experiments. Statistical significance was determined by one-way analysis of variance ANOVA and post hoc Bonferroni's multiple comparison test (^∗^*P* < 0.05).

In an effort to clarify the involvement of PP2A phosphatase in the hypoxia-mediated regulation of ERK and Akt activation, we firstly examined its mRNA level. The gene expression of PP2A was not significantly modified by hypoxia either in parental wild-type or in HIF-1 −/− cells (Figure 5(a)). Second, we employed OA, a potent inhibitor of PP2A. Before exposure to hypoxia or standard cultivation, OA was added to media in a 10 nM concentration. The inhibition of PP2A increased the phosphorylation of ERK independently of normoxic or hypoxic conditions and abolished its downregulation in hypoxia (Figures 5(b), 5(c), 5(d), and 5(e)). The phosphorylation of RAF1 at S259 remained intact upon treatment with OA (Figure 5(b)). The effect of OA on Akt phosphorylation was even more pronounced in hypoxia. Treatment with OA also increased the basal level of MEK phosphorylation, which was, however, reduced in hypoxia-cultivated samples. Similarly, OA induced the upregulation of RAF1 at S338 but did not prevent the reduction observed in hypoxic samples (Figures 5(b), 5(c), and 5(d)).

### 3.6. Hypoxia Reduces the ROS Level in ES Cells Affecting ERK and Akt Phosphorylation

**Figure 6:**
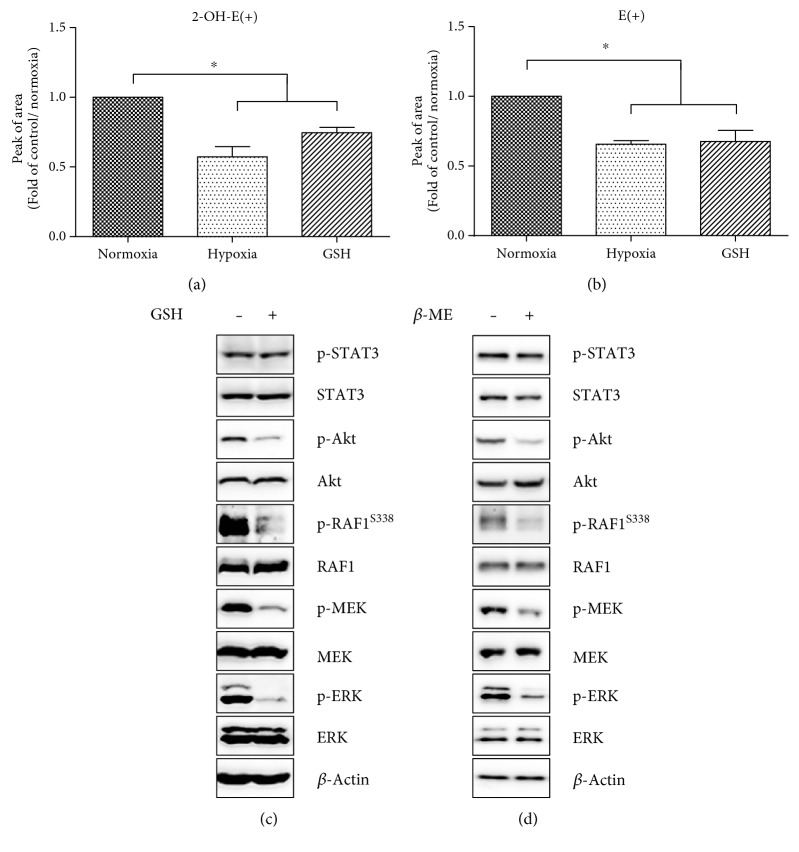
HPLC determination of specific 2-OH-E(+) (a) and nonspecific product E(+) (b) of HE oxidation in cells cultivated in normoxia or hypoxia or supplemented with GSH. Data are presented as mean + SEM from at least three independent experiments. Statistical significance was determined by one-way analysis of variance ANOVA and post hoc Bonferroni's multiple comparison test (^∗^*P* < 0.05). Effect of GSH and *β*-ME supplementation on phosphorylation of MAPK/ERK, Akt, and STAT3 (c, d). Total level of *β*-actin was used as a loading control.

To determine the involvement of hypoxia-induced changes in the cellular redox environment, we employed the ROS sensitive probe HE. Following the hypoxic cultivation, we observed a significant decline in both O_2_-specific (Figure 6(a)) and -nonspecific HE oxidation products (Figure 6(b)) as assessed by HPLC analysis. The supplementation of cells with intracellular antioxidant GSH significantly reduced the amount of HE oxidation products in a manner similar to hypoxia. Further, we sought to elucidate the effect of GSH or *β*-ME supplementation (10 mM, 60 minutes) on the phosphorylation of selected signaling components of the ERK and Akt pathways. Following the treatment with GSH or *β*-ME, we revealed robust dephosphorylation of the ERK pathway and its upstream kinases, as well as Akt kinase. The level of STAT3 phosphorylation remained unaffected by this intervention (Figures 6(c) and 6(d)).

### 3.7. Hypoxia Attenuates Markers Associated with Undifferentiated State of ES Cells

**Figure 7:**
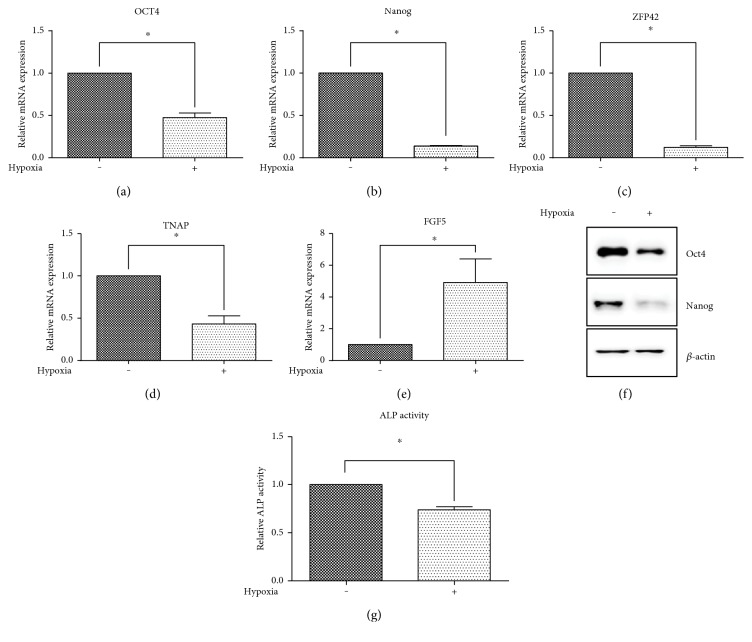
Relative expression of OCT4 (a), NANOG (b), ZFP42 (c), TNAP (d), and FGF5 (e) in wild-type ES cells determined by qRT-PCR. Data are presented as mean + SEM from at least three independent experiments. Statistical significance was determined by *T* test (^∗^*P* < 0.05). Protein level of Oct4 and Nanog in wild-type ES cells cultivated in normoxia or hypoxia as determined by western blot (f). Total level of *β*-actin was used as a loading control. Determination of alkaline phosphatase activity in wild-type ES cells cultivated in normoxia or hypoxia (g). Data are presented as mean + SEM from at least three independent experiments. Statistical significance was determined by *T* test (^∗^*P* < 0.05).

Given the suggested importance of hypoxia in stem cell cultivation, we aimed to investigate whether the attenuation of prodifferentiation ERK signaling in hypoxia affects the selected markers of stem cell maintenance. The gene expressions of critical regulators of the undifferentiated state and stem cell markers octamer-binding transcription factor 4 (OCT4), Nanog, zinc finger protein 42 homolog (ZFP42), and tissue nonspecific alkaline phosphatase (TNAP) were significantly decreased in hypoxia (Figures 7(a), 7(b), 7(c), and 7(d)), contrary to the early differentiation marker fibroblast growth factor 5 (FGF5), which was upregulated (Figure 7(e)), as determined by qRT-PCR. Protein levels of Oct4 and Nanog were reduced in hypoxia-cultivated cells as determined by western blot (Figure 7(f)), as well as alkaline phosphatase activity (Figure 7(g)).

## 4. Discussion

Properties of stem cells are maintained by numerous factors including O_2_ level. Mouse ES cells are routinely cultivated in media supplemented with serum and LIF, which support their undifferentiated state. LIF acts mainly through activating STAT3 signaling, while serum activates other pathways including MAPK/ERK and Akt [14, 16, 33]. Here, we show that chronic hypoxia (1% O_2_ for 24 h) decreases the phosphorylation of ERK and Akt but not STAT3 and attenuates markers associated with undifferentiated state. As the most striking effect of hypoxia was observed on ERK signaling, we further focused on this pathway in particular, with a minor interest also in Akt. Interestingly, chronic hypoxia downregulated not only ERK but also its upstream kinases, MEK and RAF.

As mentioned before, cellular response to hypoxia is a complex process. A decrease in O_2_ level inhibits PHD, which in turn leads to HIF stabilization and transactivation of its target genes, for example, PGK1 or VEGF [34]. GDF15 is known to be upregulated by various stressors including O_2_ deprivation, without evidence of direct HIF involvement [35, 36]. This process is universal virtually for every cell type, including ES cells.

To understand the mechanisms of the observed ERK signaling inhibition by hypoxia, we first aimed to assess whether HIF activity alone causes the dephosphorylation of ERK signaling pathways. We employed HIF-1*α* deficient ES cells, the exogenous expression of HIF-1*α* and HIF-2*α*, and treatment with the most commonly used inhibitors of PHD, (hypoxia mimetics) DMOG, JNJ, CoCl_2_, and DFO [22, 37, 38]. Neither HIF-1*α* depletion nor HIF upregulation by overexpression or hypoxia mimetics manifested an effect on the studied ERK dephosphorylation. Therefore, we suggest that hypoxia and not HIF itself is responsible for the observed attenuation of ERK signaling. Moreover, DFO and CoCl_2_ even induced ERK phosphorylation. This is in agreement with other authors reporting that iron chelation strongly activates MAPK [39–42]. Interestingly, the mechanism of ERK induction proposed by Huang and colleagues suggests that DFO inhibits DUSP1 and therefore supports ERK phosphorylation [43]. However, in our experiments, we observed the induction of DUSP1 following DFO and CoCl_2_ treatment (data not shown, manuscript in preparation). ROS-dependent activation of ERK signaling has been described multiple times [44, 45], and we earlier reported that in our system, ERK is also activated in a ROS-sensitive manner [25]. Therefore, we hypothesized that the activation of ERK might be mediated by drug-induced ROS elevation [46, 47].

Further, we aimed to establish whether the downregulation of ERK signaling is associated with the activity of phosphatases. In agreement with literature, the roles of selected MAPK-specific DUSPs and PP 2A were here analyzed. The elevated expression of DUSP1 following hypoxic treatment was reported earlier [48] as well as the upregulation of ERK-specific DUSP6 in the presence of 1% O_2_, which was mediated in an HIF-1-dependent manner [18]. We also tested DUSP5 as a representative of nuclear inducible DUSP with specificity only towards ERK; the higher efficiency of DUSP1 in the dephosphorylation of p38 and c-Jun N-terminal kinase was described by other authors [49, 50].

In our experiments, hypoxia increased the expression of DUSP1 in an HIF1-independent manner, while the mRNA expressions of DUSP5 and DUSP6 remained unchanged upon 24 h hypoxic incubation. Others suppose that DUSP1 is induced by hypoxia in a program of gene expression controlled by HIF-1 [51, 52]. Although we do not rule out this possibility, on the basis of our results, we propose that HIF-1 is dispensable for DUSP1 gene expression in chronic hypoxia. Interestingly, in contrast to our results, Bermudez and colleagues reported the upregulation of both DUSP5 and DUSP6 mRNA levels following 24 h cultivation in 1% O_2_ [18]. This divergence might be attributed to differences between the melanoma and adenocarcinoma cell lines employed in mentioned study and the ES cells used in our experiments.

Next, we employed siRNA silencing to investigate the involvement of DUSPs in the regulation of ERK phosphorylation. The downregulation of DUSP1 and DUSP5 by specific siRNAs did not have a profound effect on ERK phosphorylation. In contrary to this, the siRNA silencing of DUSP6 resulted in upregulation of the phosphorylated form of ERK, suggesting its involvement in ERK regulation in mouse ES cells. Hypoxia, however, downregulated ERK phosphorylation regardless of DUSP6 silencing. Moreover, hypoxia did not increase DUSP6 expression on either the mRNA or protein levels. It was reported that DUSP6 is subject to extensive posttranscriptional and posttranslational modifications and that mRNA level might not correspond to the protein level due to several feedback loop mechanisms that are likely to promote the proteasomal degradation of DUSP6 via ERK phosphorylation [18, 53]. Thus, it is possible that the expression of DUSP6 takes place as a part of early hypoxic response and, after 24 h, returns to its basal level. However, we observed the downregulation of DUSP6 on the protein level even after 3 h cultivation in 1% O_2_. Taken together, we conclude that selected DUSP1, 5, and 6 do not play a major role in the downregulation of ERK phosphorylation during chronic hypoxia in our system. Consistent with this is the fact that not only ERK but also its upstream kinases MEK and RAF, which are not recognized as a substrate for DUSPs [54], showed a decline in phosphorylation status in hypoxia.

Further, we decided to elucidate the role of PP2A in chronic hypoxia. PP2A is one of the most abundantly expressed serine/threonine protein phosphatases that regulates the MAPK/ERK pathway in both a positive and negative fashion [55] and is also involved in the dephosphorylation of Akt [56]. Although PP2A can be induced by various stressors including hypoxia in both in vivo and in vitro models [19, 20], in our experiments, its mRNA level was not altered after hypoxic cultivation. We employed the PP2A inhibitor OA to further investigate the involvement of PP2A in the dephosphorylation of the ERK and Akt pathways observed in hypoxia. The hypoxia-induced impairment of ERK and Akt phosphorylation was reversed in the presence of OA. The dephosphorylation of MEK was partially rescued, but it was still downregulated by hypoxia, despite the general increase in phosphorylation, similarly to the situation with the S338 RAF1 residue. These findings suggest several modes of action of PP2A in our experiments. PP2A might dominate as a negative regulator of ERK signaling through the direct dephosphorylation of ERK and MEK, as proposed by previous studies [57, 58]. However, the increased phosphorylation of S338 at RAF1 in OA-treated ES cells suggests intervention on the level of RAF1 or, even more likely, upstream, as S338 is not reported as a PP2A target. This is in accordance with the study by Sawa and colleagues [59]. Moreover, OA also promotes the phosphorylation of epidermal growth factor receptor (EGFR) and thus activates EGF signaling, which is also connected with ERK and Akt upregulation [60]. However, we were not able to detect changes in EGFR phosphorylation in our experiments (data not shown). As the inhibitory phosphorylation of RAF1 at the S259 site which is the PP2A target [61] remained unaffected by OA treatment, we also propose that this type of RAF1 regulation has negligible importance in our system. Interestingly, S259 phosphorylation is mediated by Akt [62]; however, the phosphorylation of Akt was upregulated following OA treatment in hypoxia without the effect of RAF1 phosphorylation. This suggests the limited significance of crosstalk between PI3K/Akt and MAPK/ERK at the level of Akt and RAF1 in ES cells. Taken together, our results indicate that the impairment of ERK signaling takes place on the level of RAF1 or above via a hypoxia-driven independent mechanism that might, to a certain extent, include the involvement of PP2A.

A plausible explanation for our observations might be in the decline of intracellular ROS in cells cultivated in hypoxia. ROS are currently recognized as an important modulator of various intracellular signaling pathways [63]. Many growth factors and cytokine receptors possess cysteine-rich motifs susceptible to oxidation that may result in changes in the structure and function of the protein and lead to the activation or inhibition of several signaling pathways [64]. A proportionate level of ROS is also required for the formation of disulfide bonds in order to achieve the suitable intermolecular conformation of signaling proteins and is also vital in the process of intramolecular dimerization and protein-protein interactions (e. g., with scaffold proteins), which are necessary for proper signal transduction [65].

Previously, we and other authors demonstrated that antioxidants and inhibitors of ROS-producing enzymatic sites abolish MAPK and Akt activation. Conversely, interventions that lead to increased ROS production or the direct exposure of cells to oxidants such as H_2_O_2_ also activated MAPK as well as Akt in a number of different studies including ours [25, 63, 64].

To compare the effects of hypoxia-mediated decreases in ROS, we treated cells with GSH. GSH is an essential component of the ROS buffering intracellular system and a first line of defence antioxidant [66]. Cells supplemented with exogenous GSH manifested phosphopatterns of ERK and Akt signaling identical to those of cells cultivated in hypoxia. These results were further confirmed with treatment of cells with reducing agent and thiol antioxidant *β*-ME. Therefore, we suggest that hypoxia-mediated ROS depletion is significantly involved in the downregulation of ERK and Akt signaling in conditions of chronic hypoxia.

Signaling through ERK kinase is typically regulated by mitogens and as such is associated with cell proliferation. The inhibition of mitogen-induced ERK signaling thus also attenuates cell division [67]. The described mechanisms may therefore serve as part of the processes that keep the low proliferation rate of stem cell in their niche, even in the presence of relatively high concentrations of growth factors. Multiple lines of evidence show that cultivation in the range between 1 and 5% of O_2_ supports the maintenance of ES cells in a pluripotent state, prevents their differentiation, and even reprograms the partially differentiated cells to a stem cell-like state [2, 5, 68]. In contrast to studies proposing hypoxia to have a beneficial effect on stem cell maintenance, others reported that cultivation in reduced O_2_ tension supports rather ES cell differentiation [3, 4, 69]. Although ERK signaling contributes preferably to differentiation in the context of ES cells, the hypoxia-mediated ERK attenuation observed in our experiments did not support the undifferentiated state, as shown in the reduced transcripts of markers associated with stem cell signature. This is in agreement with our earlier study in which neither PHD inhibition nor 1% or 5% hypoxia prevented the downregulation of markers associated with pluripotency induced by the depletion of LIF [22]. Here, we report similar results, even in the presence of complete medium, as shown by reduced mRNA levels of OCT4, NANOG, ZFP42, and TNAP, diminished Oct4 and Nanog protein and reduction in alkaline phosphatase activity. Thus, in our system, hypoxia is not a supportive factor with respect to the maintenance of markers of undifferentiated ES cells after 24 h cultivation in 1% O_2_. PI3K/Akt was shown to be critical for supporting the self-renewal of ES; therefore, the observed reduction in transcripts associated with undifferentiated status might be attributed to hypoxia-mediated impairment of Akt and its downstream targets such as Nanog [15, 33]. However, it should be emphasized that downregulations of markers associated with undifferentiated state are highly dependent on hypoxia level, length of hypoxic incubation, cell type, and specific culture conditions, and might have only be transient [21]. It is of particular interest that we did not observe the downregulation of STAT3 signaling in hypoxia, which is central to maintenance of the undifferentiated state and pluripotency [14, 70]. This is in contrast with a study by Jeong and collaborators, who also reported the hypoxia-induced differentiation of ES cells. However, in this study, the differentiation of ES was connected to the HIF-1-mediated suppression of LIF receptor transcription, which in turn attenuated STAT3 activation [3]. As we showed on the phosphorylation level and by luciferase reporter assay, STAT3 signaling is not compromised by hypoxia in our system, regardless of the presence of HIF-1. Notably, in our previous study, we also reported the resistance of LIF-induced STAT3 phosphorylation to changes in intracellular redox status [25]. This notion is of particular interest as persistent STAT3 phosphorylation is a hallmark of several cancers and leads to the gene expression responsible for malignant cell proliferation and resistance to apoptosis, as well as increased invasion and migration [71]. Further, it should be emphasized that STAT3 signaling is crucial for the regulation of cancer stem cells in a similar way as for the regulation of ES cells [72]. The relative indifference of STAT3 phosphorylation to changes in redox environment thus might serve as one of the driving forces of cancer progression associated with poor prognosis.

Here, we report conclusively that chronic hypoxia attenuates the phosphorylation of ERK and Akt in ES cells independently of the presence of HIF-1*α*. On the basis of our results, we suggest that ROS plays a considerable role in the phosphorylation of ERK and Akt in ES cells, as is demonstrated by the similar effect of hypoxia-induced ROS depletion and GSH supplementation on ERK and Akt signaling cascades. However, our data do not exclude the involvement of other different mechanisms.

## Figures and Tables

**Table 1:** Primers, appropriate annealing temperatures, and PCR product lengths.

Transcript	Forward 5′→3′	Reverse 5′→3′	Annealing (°C)	Product (bp)
DUSP1	tctcagccaattgtgctaac	tgatggagtctatgaagtcaa	56	126
DUSP5	cgcctacagaccagcctatg	ccttcttccctgacacagtcaat	61	264
DUSP6	acctggaaggtggcttcagt	tccgttgcactattggggtc	62	187
FGF5	accggtgaaaccaaaggtg	gcgaaacttcagtctgtacttcact	57	76
GDF15	ccgagaggactcgaactcag	ggttgacgcggagtagcag	60	104
NANOG	aagcagaagatgcggactgt	gtgctgagcccttctgaatc	59	232
OCT4	cgttctctttggaaaggtgttc	gaaccatactcgaaccacatcc	59	319
PGK1	ggtgttgccaaaatgtcgcttt	cagccttgatcctttggttgtt	58	139
PP2A	gtggatgggcagatcttctgt	gcagcttggttaccacaacg	60	347
TBP	gcacaggagccaagagtgaag	acacgtggatagggaaggca	60	397
TNAP	aggcaggattgaccacgg	tgtagttctgctcatgga	59	440
VEGF	agaaggagagcagaagtccc	gatccgcatgatctgcatgg	62	235
ZFP42	gcacacagaagaaagcagga	cactgatccgcaaacacct	57	94
*β*-actin	gatcaagatcattgctcctcct	taaaacgcagctcagtaacag	59	177
